# Quercetin Reduces Oxidative Stress and Apoptosis by Inhibiting HMGB1 and Its Translocation, Thereby Alleviating Liver Injury in ACLF Rats

**DOI:** 10.1155/2021/2898995

**Published:** 2021-10-25

**Authors:** Peng Fang, Bo Dou, Jiajun Liang, Weixin Hou, Chongyang Ma, Qiuyun Zhang

**Affiliations:** ^1^Department of Infectious Diseases, The First Affiliated Hospital of Zhejiang Chinese Medical University, Hangzhou 310006, Zhejiang Province, China; ^2^Beijing Key Lab of TCM Collateral Disease Theory Research, School of Traditional Chinese Medicine, Capital Medical University, Beijing 100069, China

## Abstract

**Background:**

Acute on chronic liver failure (ACLF) is a syndrome of acute liver failure that occurs on the basis of chronic liver disease, which is characterized by a rapid deterioration in a short period and high mortality. High mobility group box 1 (HMGB1) may be involved in the pathological process of ACLF; its specific role remains to be further elucidated. Our previous studies have shown that quercetin (Que) exerts anti-oxidant and anti-apoptotic effects by inhibiting HMGB1 in vitro. The present study aimed to investigate the effect of Que on liver injury in ACLF rats.

**Methods:**

The contents of ALT, AST, TBiL, and PT time of rats in each group were observed. HE staining was used to detect liver pathology. The levels of oxidative stress indicators such as MDA, GSH, and 4-HNE in the rat liver were detected. TUNEL assay was used to detect apoptosis in rat hepatocytes. Immunofluorescence and western blot analysis were performed to explore the protective effect of Que on ACLF rats and the underlying mechanism.

**Results:**

The results showed that Que could reduce the increase of serum biochemical indices, improve liver pathology, and reduce liver damage in ACLF rats. Further results confirmed that Que reduced the occurrence of oxidative stress and apoptosis of hepatocytes, and these reactions may aggravate the progress of ACLF. Meanwhile, the results of immunofluorescence and western blotting also confirmed that the expression of HMGB1 and extranuclear translocation in ACLF rat hepatocytes were significantly increased, which was alleviated by the treatment of Que. In addition, when cotreated with glycyrrhizin (Gly), an inhibitor of HMGB1, the inhibition of Que on HMGB1 and its translocation, apoptosis and oxidative stress, and the related proteins of HMGB1-mediated cellular pathway have been significantly enhanced.

**Conclusion:**

Thus, Que alleviates liver injury in ACLF rats, and its mechanism may be related to oxidative stress and apoptosis caused by HMGB1 and its translocation.

## 1. Introduction

Acute on chronic liver failure (ACLF) refers to a syndrome of liver failure caused by various factors on the basis of chronic liver disease, which is manifested by acute jaundice deepening and coagulopathy [1]. It can be complicated by various symptoms such as hepatic encephalopathy, ascites, infection, and extrahepatic organ failure [2]. ACLF is accompanied by rapid deterioration and high mortality in the short term. In Western countries, ACLF is mainly caused by alcoholic liver injury, improper drug use, and infection. In Asia, the main cause is hepatitis virus, and about 80% of patients are caused by acute exacerbation of HBV infection, followed by damage from drugs and hepatotoxic substances [3]. A study on hospitalized patients with liver cirrhosis in the United States showed that the incidence of ACLF among hospitalized patients with cirrhosis was 26.39%, and the 90-day mortality rate was 40.02% [4, 5]. Another 10-year cohort study conducted in China showed that the 60-day mortality rate of ACLF patients who did not undergo liver transplantation was 37.4% [6]. Even though the East and West have different understanding and definitions of ACLF, the mortality rate of ACLF is extremely high in both parties [7].

Although the pathogenesis of ACLF is poorly understood, however, the release of damage-associated molecular patterns (DAMPs) caused by immune system imbalance and inflammatory response has been confirmed to aggravate the pathological process of ACLF [8]. High mobility group box 1 (HMGB1), an evolutionarily conserved nuclear DNA binding protein, is widely present in eukaryotic cells and has important biological activities both inside and outside the cell [9, 10]. Once released outside the cell membrane, it can also act as DAMP. Many convincing evidence indicate that the pathological process of ACLF is affected by HMGB1 [11, 12].

Quercetin (3,3′,4′,5,7-pentahydroxyflavone, Que), a typical flavonol-type flavonoid, is also considered as a potential inhibitor of HMGB1 [13]. Several research suggest that Que has a wide range of biological effects such as anti-oxidant, anti-inflammatory, and anti-apoptosis [14, 15]. Supplementing the diet with Que has beneficial effects on many liver diseases [16]. Que improves liver cell damage by inhibiting inflammation, oxidative stress, and cell apoptosis, thereby reducing liver damage caused by various hepatoxins in vivo [17–19]. As an effective phytochemical component for the treatment of various liver diseases, it has been studied in hepatitis, acute liver failure, and fibrosis [13, 20, 21]. Our previous studies have shown that Que exerts anti-oxidant and anti-apoptotic effects via inhibiting HMGB1, thereby protecting the liver cell from damage caused by D-GaLN in vitro [22]. In the present ACLF rat model, the application of D-GaLN is the main stimulating factor for acute liver injury. However, whether Que can reduce liver injury in ACLF rats has not yet been adequately studied. In the present study, we investigated the protective effect of Que on liver injury in ACLF rats, following the research method of the hepatoprotective effect of flavonoids [23]. In addition, an exact inhibitor of HMGB1 was combined to further verify the hypothesis that HMGB1 plays an important role in the disease process of ACLF and the beneficial therapeutic effect of its inhibition.

## 2. Materials and Methods

### 2.1. Chemicals and Regents

Que was obtained from Sigma-Aldrich (St. Louis, USA; cat: Q4951); its purity is ≥95%. Human serum albumin (HSA; cat: A9731), D-galactosamine (D-GaLN; cat:G1639), and lipopolysaccharides (LPS; cat:L3012) were also obtained from Sigma-Aldrich (St. Louis, USA). Anti-Bcl-2 (cat: ab19645), anti-Bax (cat:ab32503), anti-HMGB1 (cat:ab79823), anti-iNOS (cat:ab49999), anti-COX-2 (cat:ab15191), and anti-4HNE (cat:ab48506) were obtained from Abcam (Shanghai, China). Anti-TLR-4 (cat: SC-293072) was obtained from Santa Cruz Biotechnology (Santa Cruz, USA). Anti-caspase-9 (cat: #9508), anti-caspase-3 (cat: #9662), anti-NF-*κ*B p65 (cat:#8242) were obtained from Cell Signaling Technology (Boston, USA).

### 2.2. Experimental Animals

Ninety male Wistar rats weighing 200 to 240 g were purchased from Vital River Laboratory Animal Technology Co. Ltd. (Beijing, China). The animals were housed in a specific pathogen-free environment under constant temperature (25 ± 3°C) and humidity (60 ± 10%), with a 12 h light/dark cycle. All animals were acclimated to the environment for 5 days before the experiments. All of the procedures were performed according to the Institutional Guidelines for the Care and Use of Laboratory Animals and were authorized by the Animal Ethics Committee of Capital Medical University (NO.AEEI-2019-067).

### 2.3. Animal Treatment

**Figure 1:**
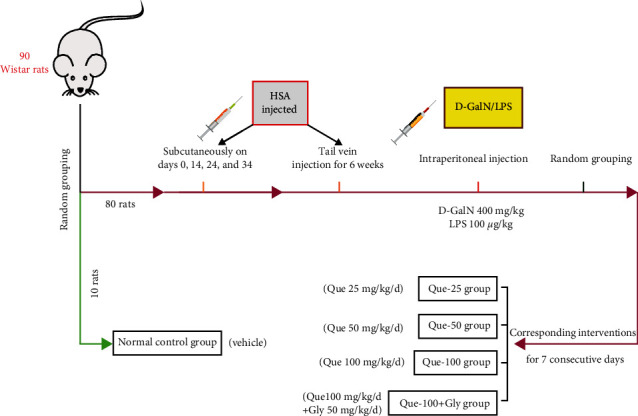
Establishment of ACLF rat model and experimental intervention. The rats were injected with HSA to induce immune hepatic fibrosis. At first stage, rats were sensitized by subcutaneous injection of HSA solution (0.5 ml, HSA 4 mg) for a total of 4 injections (days 0, 14, 24, and 34). Subsequently, tail vein injection was performed twice a week for 6 weeks (0.5 ml, gradually increased the HSA dose, 2.5 mg⟶3 mg⟶3.5 mg⟶4 mg⟶4.5 mg, and then maintained at 4.5 mg), and the normal group was injected with the same amount of normal saline. And then, intraperitoneal injection of 400 mg/kg D-GaLN and 100 *μ*g/kg LPS caused acute liver injury to establish the ACLF model. Finally, the rats were randomly divided into 5 intervention groups: receiving Que and/or glycyrrhizin, or vehicle treatment for 7 consecutive days. The normal control group underwent the same procedures without therapeutic intervention.

The ACLF rat model was established as we described previously [24]. Briefly, acute liver failure was induced on the basis of chronic immune liver fibrosis. As shown in (Figure 1), except for the normal control group (*n* = 10), the remaining 80 rats were injected with HSA to induce immune liver injury. After 6 weeks, 50 survived rats with liver fibrosis confirmed by Masson's trichrome staining [25] were selected, and then the rats were injected intraperitoneally with 400 mg/kg D-GaLN and 100 *μ*g/kg LPS to establish the ACLF model. Then the rats were randomly divided into 5 groups: (1) ACLF group, rats were intragastric administration of an equal volume of normal saline solution and intraperitoneal injection of an equal amount of vehicle; (2) low-dose Que treatment group (Que-25), rats were intragastric administration of 25 mg/kg Que for 7 consecutive days and intraperitoneal injection of an equal amount of vehicle; (3) middle dose of Que treatment group (Que-50), treatment was the same as the Que-25 group, while the dose of Que was 50 mg/kg; (4) high dose of Que treatment group (Que-100), treatment was the same as the Que-25 group, while the dose of Que was 100 mg/kg; and (5) HMGB1 inhibitor intervention group (Que-100 + Gly), rats were intragastric administration of 100 mg/kg Que and intraperitoneal injection of 50 mg/kg glycyrrhizin (Gly) for 7 consecutive days. Gly is a direct inhibitor of HMGB1, which can bind to HMGB1 directly, interacting with two shallow concave surfaces formed by the two arms of both HMG boxes [26, 27]. At the end of the experiment, there were 10 survivors in the normal control group, 5 in the ACLF group, 6 in Que-25 group, 6 in the Que-50 group, 7 in the Que-100 group, and 7 in the Que-100 + Gly group. Before tissue collection, rats were deeply anesthetized by intraperitoneal injection of 1% pentobarbital sodium (40 mg/kg). After the anesthesia was stable, blood was collected from the abdominal aorta, and the serum collected by centrifugation was stored at −80°C. The liver tissue was quickly collected and weighed, frozen in liquid nitrogen, and stored at −80°C. Then the rats were euthanized by cervical dislocation.

### 2.4. Determination of Serum Biochemical Indices

Blood samples were collected in tubes and centrifuged for 15 min at 3,000 rpm (Sigma-Aldrich, USA) to collect serum. The levels of alanine aminotransferase, aspartate aminotransferase (AST), and total bilirubin (TBiL) in serum were detected with an automatic analyzer (Hitachi, Inc., Japan) using commercial kits following the manufacturer's instructions.

### 2.5. Determination of Prothrombin Times

Blood samples were collected in anti-coagulant tubes containing sodium citrate solution and centrifuged for 15 min at 3,000 rpm (Sigma-Aldrich, USA) to collect plasma. Prothrombin times (PTs) were measured using a kit (Nanjing, China) according to the manufacturer's instructions.

### 2.6. Liver Histological Observation

Left lobes of liver tissues were isolated and fixed immediately with 10% neutral buffered formalin. The paraffin-embedded liver tissue samples were cut into 5 *μ*m thick sections for hematoxylin and eosin (H&E) staining, and then the sections were observed with a pathological section panoramic scanner (Leica Aperio AT2).

### 2.7. Assessment of Oxidative Stress

The content of hepatic malondialdehyde (MDA) was determined by thiobarbituric acid (TBA) reagent test using a commercial kit (Beyotime, China; cat: S0131). The liver homogenate was mixed with TBA buffer, incubated at 95°C for 1 hour, and then incubated on ice to stop the reaction. The mixture was centrifuged (4,000 rpm; 10 min), and the absorbance was measured by a microplate reader at a wavelength of 532 nm. The results were presented as nmol/mg protein.

The level of anti-oxidant enzyme-reduced glutathione (GSH) content was determined by the 5,5'-dithiobis-(2-nitrobenzoic acid) (DTNB) reactant test using a commercial kit (Beyotime, China; cat: S0053). Briefly, after mixing liver homogenate with DTNB stock solution and reacted, the absorbance was measured at a wavelength of 412 nm by a microplate reader. The GSH content in the sample was calculated according to the standard curve and presented as nmol/mg protein.

### 2.8. Immunofluorescence Analysis

Briefly, after dewaxing and antigen retrieval, the paraffin section was blocked by incubating with bovine serum albumin (BSA). Then the sections were individually incubated with anti-4-hydroxynonenal (4-HNE), anti-TLR-4, and anti-HMGB1 at 4°C overnight. After washing with PBS, the sections were incubated with FITC or TIRTC-labeled secondary antibody for 2 h at 37°C in the dark. Then the sections were washed 3 times with PBS for 5 min each time. Then, the tables were sealed with antifluorescence attenuation sealing solution (containing DAPI). Fluorescence images were collected by using a confocal microscope (Leica TCS SP8), and the results were analyzed using Image J software version 1.80.

### 2.9. Terminal Deoxynucleotidyl Transferase dUTP Nick End Labeling (TUNEL) Assays

The apoptotic response of hepatocytes was detected with paraffin-embedded sections using a TUNEL assay and Fluorescein In Situ Cell Death Assay Kit (KeyGEN BioTECH, China; cat: KGA7072) according to the manufacturer's instructions. The positive cells were counted in 10 random fields at 400X magnification, and 3 sections of each sample were analyzed.

### 2.10. Western Blot Analysis

Liver proteins were homogenized and then collected by using RIPA lysis buffer. Cytoplasmic and nuclear proteins were isolated using nuclear and cytoplasmic protein extraction kits (Beyotime, China; cat: P0028), according to the manufacturer's instructions. The BCA protein assay reagent kit was used to determine the concentration of total liver protein and the extracted nuclear protein and cytoplasmic protein. An equal amount of protein (30 *μ*g) was separated by 8–12% SDS-PAGE and transferred into PVDF membranes. Next, membranes were incubated with Tris-buffered saline, containing 5% non-fat dry milk for blocking purposes at room temperature for 1 hour. Then, membranes were incubated overnight at 4°C with primary antibodies directed against HMGB1, TLR-4, caspase-3, caspase-9, Bax, Bcl-2, NF- kB p65, iNOS, and COX-2. After washing with TBST, the membrane was incubated with a secondary antibody for 1 h at room temperature. Finally, the reaction was detected with an enhanced chemiluminescent reagent (NCM Biotech, China; cat: P10100). An ImageQuantLAS4000 chemiluminescence imaging system was used to visualize the target proteins (GE Co., USA), and densitometry was performed using the Image J software version 1.80.

### 2.11. Statistical Analysis

All data in the present study were analyzed using Prism 8.0 and expressed as the mean ± standard deviation (SD). Differences between groups were determined by ANOVA with Tukey's post hoc test. *p* < 0.05 was regarded as statistically significant.

## 3. Results

### 3.1. Que Alleviates Hepatic Injury in ACLF Rats

**Figure 2:**
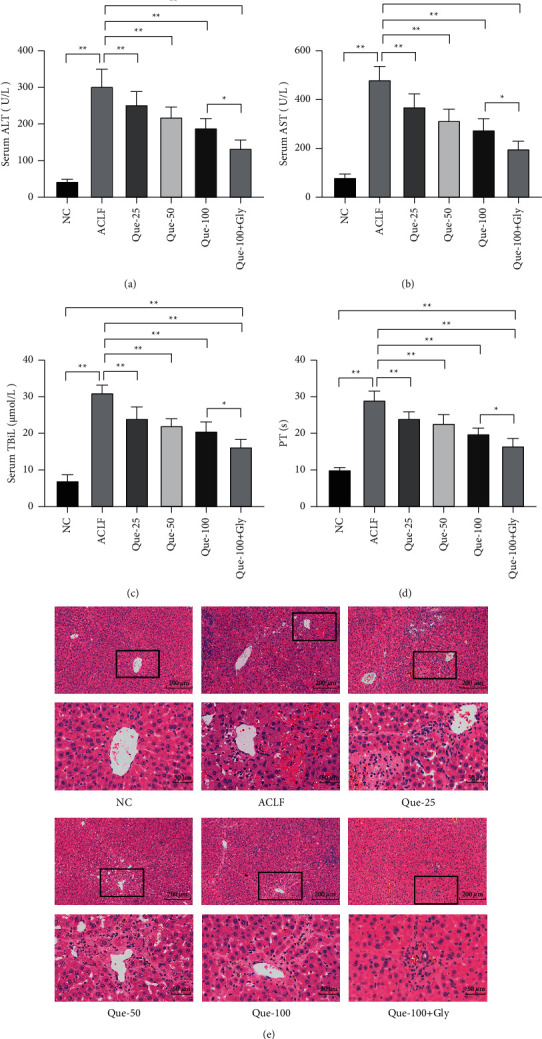
Effects of different doses of Que on liver function and pathology in acute on chronic liver failure (ACLF) rats: (a) the serum levels of alanine aminotransferase, (b) aspartate aminotransferase (AST), (c) total bilirubin (TBiL), (d) prothrombin times (PTs), and (e) hematoxylin and eosin (H&E) staining. Magnification 200X and 800X; scale bar: 200 *μ*m and 50 *μ*m; data are presented as the mean ± SD (^*∗*^*p* < 0.05, ^*∗∗*^*p* < 0.01, representative of 5–10 rats/group).

As shown in Figures 2(a)–2(d), serum ALT, AST, and TBiL were significantly increased, whereas PT was significantly prolonged in the ACLF model group, and these increases were attenuated dose dependently by Que. Furthermore, H&E staining was performed to verify the extent of liver injury. In the normal control group, clear lobular structures could be observed, and hepatocytes were arranged in an orderly manner. In the ACLF group, disordered cell arrangement, inflammatory cell infiltration, hepatic sinus expansion and bleeding, and numerous necrotic liver cells were observed. However, the treatment with Que at the dose of 25 mg/kg, 50 mg/kg, and 100 mg/kg ameliorated liver pathological damage, and the dose of 100 mg/kg Que was more obvious (Figure 2(e)). On the basis of the results of liver function and pathological analysis, 100 mg/kg Que was chosen as the optimal dose for further studies. What's more, when compared with Que-100, the ALT, AST, TBiL, and PT were further decreased after addition with Gly, an inhibitor of HMGB1, and the amelioration of pathologies showed the same performance.

### 3.2. Que Reduces Oxidative Stress Damage in ACLF Rats

**Figure 3:**
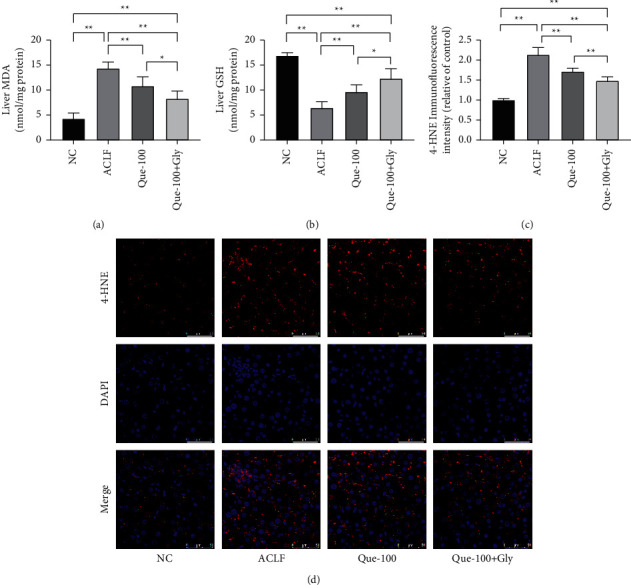
Effects of Que on oxidative stress damage in ACLF rats. The content of hepatic malondialdehyde (MDA); (b) glutathione (GSH); (c, d) immunofluorescence analysis of 4-hydroxynonenal (4-HNE). Magnification 400X; scale bar: 50 *μ*m; data are presented as the mean ± SD (^*∗*^*p* < 0.05, ^*∗∗*^*p* < 0.01, representative of 5–10 rats/group).

To assess the oxidative stress damage, the levels of MDA and GSH in the liver of rats were detected. The MDA level (Figure 3(a)) was significantly increased, and the GSH level (Figure 3(b)) was decreased in the ACLF group. However, the intervention of Que reduced the increase in MDA and increased the level of GSH. The level of 4-HNE accumulation, the main product of lipid peroxidation [28], was measurement by IF. Massive 4-HNE accumulation was in hepatocytes of the ACLF group, which decreased after Que intervention. What's more, the above-mentioned effects of Que were significantly enhanced by Gly (Figures 3(c) and 3(d)).

### 3.3. Que Inhibits Hepatocyte Apoptosis in ACLF Rats

**Figure 4:**
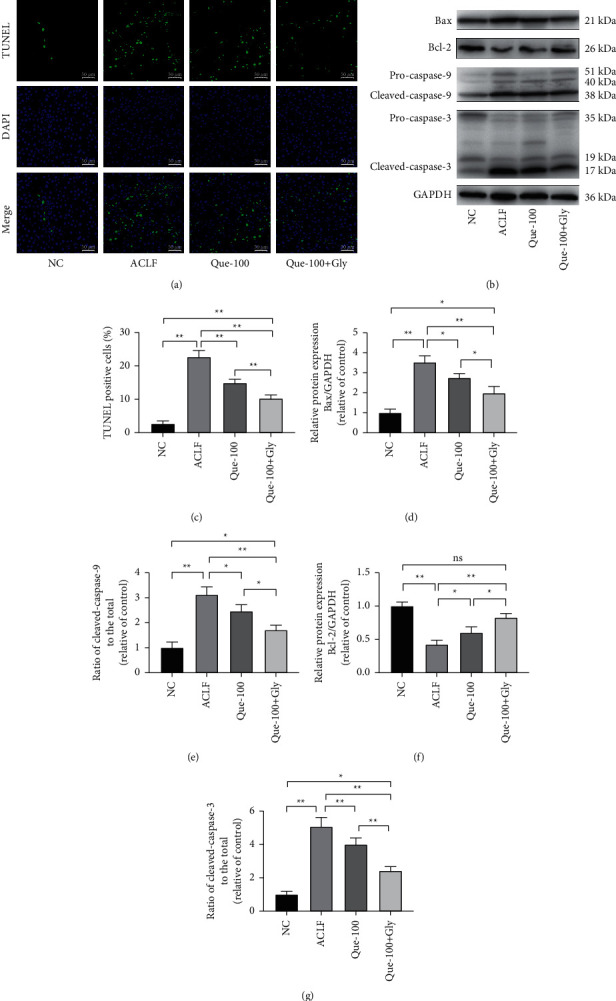
Effects of Que on apoptosis in ACLF rats. (a, c) Representative stainings and positive cells of TUNEL assays. The positive cells were counted in 10 random fields at 400X magnification, and 3 sections of each sample were analyzed, representative of 5–10 rats/group, scale bar: 50 *μ*m. (b, d, e, f) Representative western blot analyses of apoptosis-related proteins (Bax, Bcl-2, Pro-caspase-9, caspase-9, Pro-caspase-3, and caspase-3). Data are presented as the mean ± SD (^*∗*^*p* < 0.05, ^*∗∗*^*p* < 0.01). The blots shown are representative of 3 independent experiments.

Next, the extent of apoptosis in liver tissues was evaluated by TUNEL staining, which labels 3′-OH ends of DNA by ribonuclease that are activated during apoptosis. Our results showed that the number of TUNEL-positive cells in the ACLF group dramatically increase, while Que blocked the changes significantly (Figures 4(a) and 4(c)). Furthermore, we performed western blot to detect changes in apoptosis-related proteins. As results (Figures 4(b) and 4(d)–4(g)) shown, the upregulation of Bax, the ratio of cleaved caspase-9 and cleaved caspase-3, and the downregulation of anti-apoptotic protein Bcl-2 were observed in the ACLF group, which were reversed by Que treatment. Moreover, after addition with Gly, this anti-apoptotic effect was enhanced.

### 3.4. Que Decreases the Expression and Translocation of HMGB1 in Hepatocytes of ACLF Rats

**Figure 5:**
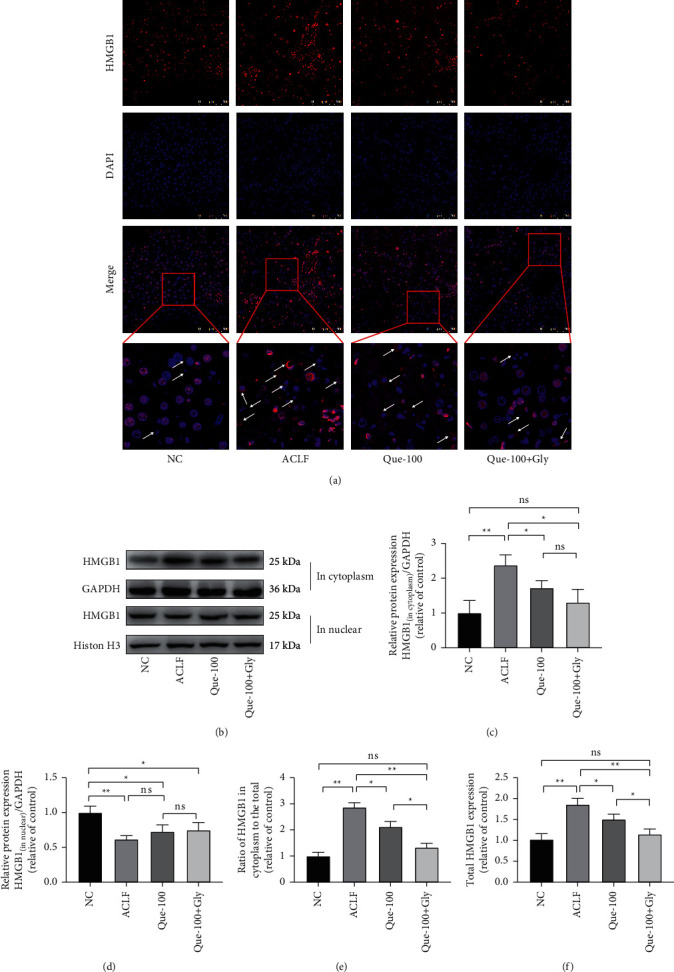
Effects of Que on the expression and translocation of HMGB1 in ACLF rats. (a) Immunofluorescence staining of HMGB1 expression and translocation. Arrows indicate the HMGB1 in cytoplasm. Magnification 400 (X); scale bar: 100 *μ*m. (b) Representative immunoblots for the HMGB1 in the nucleus; HMGB1 in the cytoplasm. (c, d) HMGB1 in the cytoplasm and nucleus under different treatments under different treatment by western blot assay. (e, f) Calculated results of the ratio of HMGB1 in the cytoplasm and the total expression of HMGB1. According to the different positions of HMGB1 expressed in the cytoplasm and nucleus, GAPDH and histone H3 were selected as housekeeping proteins. Data are presented as the mean ± SD (^*∗*^*p* < 0.05, ^*∗∗*^*p* < 0.01). The blots shown are representative of 3 independent experiments.

On the basis of our previous research, Que could inhibit HMGB1-mediated hepatocyte damage in vitro [22]. Therefore, in order to determine whether the improvement effect of ACLF by Que is related to HMGB1, we performed IF and western blot to detect the expression of HMGB1. IF showed the increased expression and distribution in the cytoplasm of HMGB1 in the ACLF group (Figure 5(a)). Western blot also confirmed that the total amount of HMGB1 and the ratio of HMGB1 in the cytoplasm to the total were increased (Figures 5(b)–5(f)). The treatment of Que reduced the increase and translocation of HMGB1. While cotreated with Gly, the inhibition was significantly enhanced.

### 3.5. Que Inhibits HMGB1-Mediated Signaling Pathway

**Figure 6:**
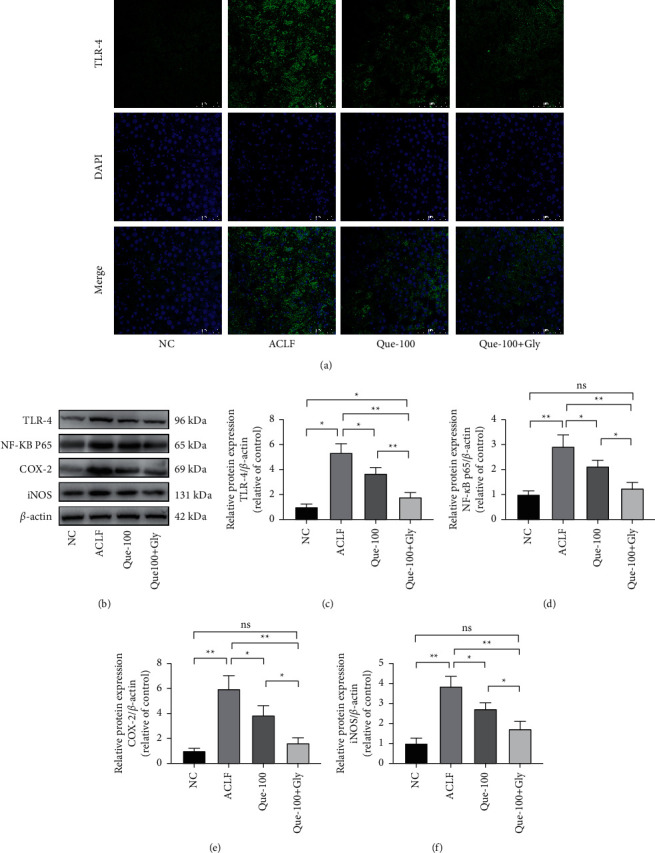
Effects of Que on the HMGB1 signaling pathway. (a) Immunofluorescence staining of TLR-4 receptor expression under different treatment conditions. Magnification 400X; scale bar: 75 *μ*m. (b–f) The TLR-4, NF-*κ*B P65, iNOS, and COX-2 proteins expression levels were evaluated by western blot assay. Data are presented as the mean ± SD (^*∗*^*p* < 0.05, ^*∗∗*^*p* < 0.01). The blots shown are representative of 3 independent experiments.

Next, to investigate the molecular mechanism of Que on HMGB1-mediated oxidative stress and apoptosis in ACLF, we analyzed changes in proteins expression of related pathways. The expression of TLR-4, an HMGB1 receptor, was significantly increased (Figures 6(b) and 6(c)), and IF showed the extensive expression of TLR-4 in the cytoplasm of damaged hepatocytes. The treatment of Que reduced this kind of expression (Figure 6(a)). Moreover, the expressions of related pathway proteins NF-kB-p65, iNOS, and Cox-2 were also increased in the ACLF group, and the treatment of Que reduced this increase of expression. What's more, the cotreatment of Gly, the inhibition effect on the expression of TLR-4, and related pathway proteins were significantly enhanced over that of Que alone (Figures 6(b) and 6(d)–6(f)).

## 4. Discussion

At present, the pathophysiology of ACLF remains poorly understood, and pharmacological approaches to reduce mortality from ACLF are still lacking. However, increasing evidence indicate that HMGB1 may be involved in the pathological progress of liver failure [11, 29]. A study on the detection of hepatocyte death biomarkers in patients with hepatitis B virus-related ACLF (HBV-ACLF) finds that the serum HMGB1 level of HBV-ACLF patients is significantly higher than that of healthy controls and chronic hepatitis B (CHB) patients [30]. Moreover, the increased expression of HMGB1 is significantly correlated with the occurrence of ACLF [31]. A meta-analysis also indicates that HMGB1 may be a useful therapeutic target for severe hepatitis B and ACLF [32]. Meanwhile, the translocation of HMGB1 to extranuclear does not exist in hepatocytes of healthy people and CHB patients. But, in ACLF patients, even in their non-necrotic hepatocytes, a lot of extranuclear translocations occurred. The nucleus-to-cytoplasm translocation of HMGB1 is a key process prior to its extracellular secretion [33].

The extracellular HMGB1, which acts as a DAMP factor, plays an important role in various liver injuries. Especially in severe liver injury, the level of HMGB1 is significantly increased [34]. However, previous studies have focused more on the proinflammatory effects of HMGB1. The increasing credible evidence confirms that HMGB1 is also essential to mediate the occurrence of oxidative stress [35]. In vitro, recombinant HMGB1 caused oxidative stress with TLR-4-dependent activation of NADPH oxidase [36]. What's more, HMGB1 activates the TLR-4 signal transduction pathway and induces the translocation of NF-*κ*B-p65 subunits to the nucleus, thereby increasing its transcriptional activity [37]. Thus, the activation of COX-2 and iNOS is induced, leading to the accumulation of 4-HNE, causing lipid peroxidation and oxidative stress [38, 39].

For liver failure, excessive apoptosis is also one of the main ways of cell death, which is also confirmed in our current experiment. And, the release of HMGB1 is also present in apoptotic cells. HMGB1 can be released in late apoptotic cells by binding to DNA [40]. Macrophages are also activated by apoptotic cells to release HMGB1 [41]. After being released, caspase-3 dependent apoptosis can be activated by HMGB1 through the TLR-4 pathway [42]. Moreover, it has been confirmed that blocking HMGB1 can inhibit caspase-3 activation, thereby reducing cell apoptosis [43]. Oxidative stress regulates the mitochondrial membrane potential, leading to the initiation of apoptosis in the mitochondrial pathway [44]. Mitochondria plays an important role in apoptosis by relocating intermembrane mitochondrial proteins, such as Bcl-2 and Bax [45]. Here, in the present study, we found that HMGB1 may play a regulatory role in hepatocyte apoptosis and oxidative stress in ACLF rats. Therefore, we hypothesize that HMGB1-mediated apoptosis is caused by the mitochondrial release of apoptotic proteins caused by oxidative stress. To our best knowledge, this mechanism by which HMGB1 is involved in ACLF pathological progression is confirmed for the first time.

**Figure 7:**
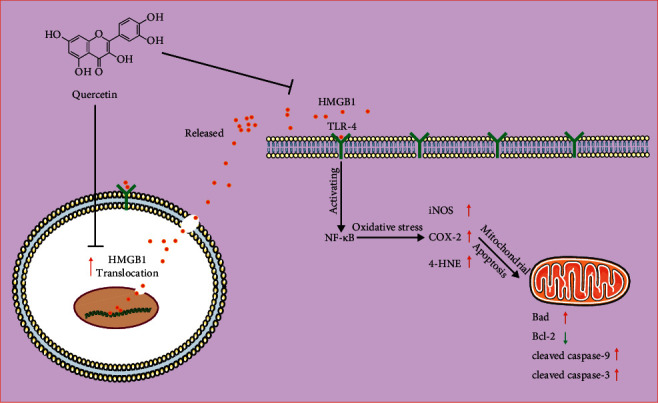
The mechanism of Que attenuating liver injury in ACLF rats by inhibiting HMGB1 and its translocation.

Que, as an effective phytochemical ingredient for the treatment of various liver diseases, has been proved to have hepatocellular protection in vivo and in vitro [46]. Que inhibits the production of oxidative markers and the activation of NF-*κ*B and MAPK signaling pathways; thus, the expression of apoptosis-related proteins has been induced in acute liver failure (ALF) mice induced by LPS/D-GalN [21]. Que also inhibits the translocation and release of HMGB1 in macrophages induced by LPS and protects mice from immune liver injury induced by Con-A by inhibiting the HMGB1-TLR2/TLR4-NF-*κ*B pathway [20]. Our previous research shows that Que inhibits HMGB1-mediated oxidative stress and apoptosis, thereby protecting L02 cells from D-GaLN mediated damage in vitro [22]. In the present study, we confirmed that Que could reduce the pathological damage, the occurrence of oxidative stress, and apoptosis in ACLF rats for the first time. The treatment of Que also reduced the translocation and overexpression of HMGB1, and its signaling pathway proteins mediated by it. The cotreatment with Gly, a direct HMGB1 inhibitor, further inhibited HMGB1 and its translocation, as well as the oxidative stress and apoptosis mediated by it, when compared with Que alone. Therefore, part of the mechanism of Que attenuating ACLF may be related to inhibiting HMGB1 and its translocation, thereby the oxidative stress and apoptosis mediated by it (Figure 7). However, there are some limitations in current research, such as the effect of Que on ACLF rats after HMGB1 overexpression or activation was not observed, and also the lack of a group with Gly alone. These should be considered in our future research.

## 5. Conclusion

In conclusion, our present study confirmed that HMGB1 and its translocation were involved in ACLF, and the specific mechanism may be related to the oxidative stress and apoptosis mediated by it. Thus, this provides further evidence for ACLF treatment with intervention HMGB1 as the target. And also Que may provide a new pharmacological intervention option for ACLF.

## Data Availability

The data used to support the findings of this study are available from the corresponding author on reasonable request.
